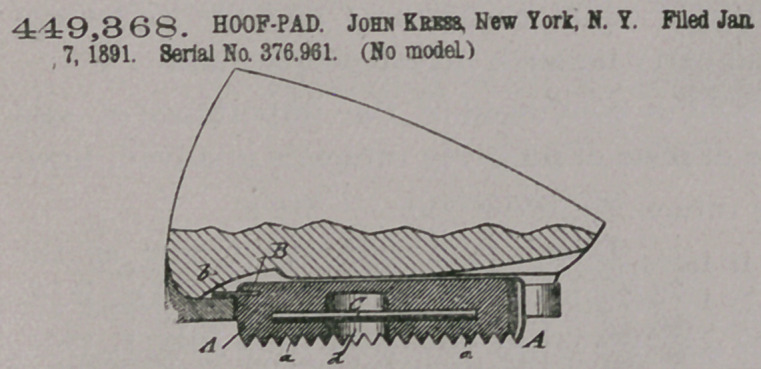# Recent Patents

**Published:** 1891-05

**Authors:** 


### RECENT PATENTS

RELATING TO

### VETERINARY MEDICINE AND ANIMAL INDUSTRY.

*Issued by U. S. Patent Office for Month ending April,* 1891.

**448,256. AUTOMATIC ANIMAL-FEEDER.** LUCIUS PERSON, Holyoke, Mass. Filed May 31, 1890. Serial No. 353,780. (No model.)

*Claim.*—In an automatic feed-box for animals, the combination of a winding-key *h* and cord-winding pulley *i*, combined and adapted to be made fast to the alarm-winding shaft of a clock, a cord *m*, secured to pulley *i*, cords *n n*, attached to cord *m*, eyes *p* and *r* for guiding cords *n n*, and doors H H, hinged in boxes I I and inclined therein to have their lower free edges attached to cords *n n*, passing thereto through the wall of the boxes, all operating as and for the purpose set forth.

**448,262. APPARATUS FOR WATERING HOGS.** GEORGE F. RUNNELS, Waco, Nebr. Filed Jan. 7, 1891. Serial No. 377,017. (No model.)

*Claim.*—1. The combination, with the tank and inlet-pipe, of the recessed float hinged within the tank and having plate I1 within the recess, the valve in the inlet-pipe, the rod F, connected to the valve and having bent end H1, and the spring E, acting on said valve and arranged within the inlet-pipe, substantially as described.

2. The combination, with the tank and the inlet-pipe, of the recessed float hinged within the tank and extended at right angles to the inlet-pipe with the recess opposite the end of said pipe, the adjustable right-angled plate I1, secured in said recess, the valve in the inlet-pipe, the bracket G in the inlet-pipe, passed through one end of the valve and having vertical arm G1, the spring E, held between said arm and the valve, and the rod F, connected at one end to the valve and the other end bent upward at right angles to its length and arranged to engage the plate I1, substantially as shown and described.

**448,736. HOOF-SPREADING HORSESHOE.** WILLIAM TOBY, Guysborough, Canada. Filed May 29, 1890. Serial No. 353,663. (Model.)

*Claim.*—The plates E E, integrally connected at the rear and provided in front with the calk D and clip F, in combination with the two pivoted sections A A and the two-armed spring C, the latter held by a pivot G in the space between the plates E E and sections A A, substantially as shown and described.

**448,330. DEVICE FOR CONTROLLING HORSES.** EDWIN J. FRASER, San Francisco, Cal. Filed Nov. 20, 1890. Serial No. 372,081. (No model.)

*Claim.* A device for controlling horses, consisting of an endless girdle surrrounding the nose and chin of a horse, a spring or other elastic strip adjustably connected with the front of the girdle so as to rest upon the bridge of the horse's nose, pads fixed to the ends of said spring and retained by it out of contact with the nasal passages, suspending and adjusting straps by which the device is supported from the headstall and throat-latch, and a bit independently connected with the headstall and with the driving reins, whereby a pull upon the reins will act to force the mouth open and through the opening of the mouth cause the spring to be bent and the pads to press upon the nasal passages, substantially as herein described.

**448,312. DEVICE FOR ATTACHING HORSESHOES.** RICHARD BARCLAY, Sodom, Ohio. Filed May 17, 1890. Serial No. 352,165. (No model.)

*Claim.*—1. The combination, with a nailless horseshoe 1, of a quarter-boot 2, connected with the shoe and formed in two parts or sections 5 and 6, and screw-bolts 14, passed through the rear boot-sections 6 and adapted to bear against the hoof to tighten the boot, substantially as described.

2. The combination, with a nailless horseshoe 1, of a quarter-boot 2, formed in two parts 5 and 6, hinged together on one side and provided with flanges 3 and lugs 9, the bolts 4, passed through said shoe and flanges, the bolt 8, engaging the lugs 9 to fasten the quarter boot, and the bolts 14, passed through the rear boot section 6 and adapted to bear against the hoof to tighten the boot, substantially as described.

3. The combinaiion, with a naillsss horseshoe 1, of the quarter-boot 2, formed in two parts hinged together on one side, the stocking 16, and the bolts 14, passed through the rear section of the quarter-boot to bear against the hoof and tighten the boot, substantially as described.

**448,366. HORSESHOE-GAGE.** WILLIAM C. PRICE, Woodburn, Oreg. Filed Aug. 1, 1890. Serial No. 360,672. (No model.)

*Claim.*—1. In a gage of the class described, the combination, with a base having a series of radiating studs and adjusting-disks mounted on the studs, of a flexible gage-strip mounted within and embraced by the studs, substantially as specified.

2. In a gage of the class described, the combination, with a central base, of a series of radiating studs, friction-disks mounted on the studs and a flexible gage-strip curved and and inserted between the disks and having its upper edge extending above the base, substantially as specified.

3. In a gage of the class described, the combination, with a central base, a series of radiating studs, and a series of adjusting-disks mounted on the studs, of a flexible gage-strip longitudinally slotted to loosely receive the studs and mounted between the sides of the base and the inner faces of the disks, substantially as specified.

4. In a gage of the class described, the combination with a central horseshoe-shaped disk, opposite heel, toe, and intermediate radiating studs and disks mounted for sliding upon and having frictional contact with the studs, of a flexible gage-strip provided with opposite longitudinal slots extending from each side of its centre to near the extremities, said slots receiving the heel and intermediate studs and provided with a central opening for the reception of the toe-stud, substantially as specified.

5. In a gage of the class described, the combination, with a central base having radiating studs, adjusting disks mounted on the studs, and a flexible gage-strip bent and sprung between the studs, of a gage sleeve or loop mounted upon one of the terminals of the strip, substantially as specified.

6. In a gage, the combination, with the base and an encircling flexible gage-strip, of combined strip supporting and adjusting devices extending from the base, substantially as specified.

**[illegible]8,546. ANIMAL-COLLAR.** GUSTAV R. SAGELSDORFF, Medford, Wis. Filed Oct. 1, 1890. Serial No. 366,729. (No model.)

*Claim.*—1. In a chain adapted to serve as an animal-collar, the combination, with two chain-sections, of the U-shaped middle piece which connects them, a pin K, supported in the outwardly-curved end of said middle piece, and a cow-bell hook turning loosely on said pin, substantially as shown and described.

2. A chain adapted to serve as an animal-collar, the same being provided with a stud F, having a reduced neck or shank and secured to one of the terminal links and adapted to pass through the other terminal link, a hook attached to said stud, and the link H, secured to the chain contiguously to the stud, and thus adapted for engagement with the aforesaid hook to form the lock hereinbefore described.

**448,557. SHEEP-SHEARS.** JOHN W. STROTHER, Cherry Camp W. Va. Filed Apr. 9, 1890. Serial No. 347,199. (No model.)

*Claim.*—The herein-described improved animal-shears, comprising, in combination, the shank 1, having the flattened portion 7, formed with tines 8, having flat upper sides, cutting-edges 9, straight vertical sides 9a, downwardly and inwardly bevelled or inclined sides 9b, and shoulders 10, having blunt points 11, the shank 2, having the shearing blade 12, the connecting-spring 5, and the hook or stop 13, all constructed and arranged substantially as and for the purpose herein set forth.

**449,485. HORSE-TRAINING BLIND.** Brewster A. Long, Troy Pa. Filed Oct. 4, 1890. Serial No. 367,132. (No model)

*Claim.*—1. In a training-blind, a main portion consisting of a straight longitudinal piece curved transversely, provided at its lower edge with a rearwardly-projected padded flange or supporting piece having its rear edge conformed to the contour of the front side of the animal's head just below the eyes and arranged and adapted at such rear edge to bear snugly against the animal's head, and provided with devices by which it may be secured in place, all substantially as and for the purposes set forth.

2. A training-blind, substantially as described, having a main piece or portion provided at its lower edge with a rearwardly-projected flange or portion and having along the edge of such flange or portion a cushion of fibrous-like material, whereby to fit closely against the face of the animal, and devices by which to secure the blind in place, substantially as set forth.

3. The improved training-blind herein described, consisting of the main portion A, made straight longitudinally and bent or curved laterally, the flange piece or portion B projected rearwardly from the lower edge of the piece A and having its rear edge conformed generally to the contour of the horse's face shortly below the eyes, the fibrous-like cushion secured along the said rear edge, the straps C, having loops C1, and the straps D, all substantially as and for the purposes set forth.

**449,488. HORSE-COLLAR.** Silas T. Marlette, Niagara Falls, N. Y. Filed May 3, 1890. Serial No. 350,544. (No model.)

*Claim.*—1. An improved horse-collar provided at the ends of its sides with connection-pieces C and D, the connection D being provided with a socket *g* and a hook E, and the connection C being provided with a projection G to enter socket *g* and with a bearing F for engagement by the hook E, all substantially as and for the purposes set forth.

2. In a horse-collar, the combination of the connection-piece C, provided with a bearing F, the connection-piece D, and the fastening-bar pivoted to said piece D and provided with a hook E and side lugs *e*, substantially as set forth.

3. In a horse-collar, the combination of the connection-piece D, having a socket *g*, and connection-piece C, having a bearing F and a projection G to enter the socket *g*, the fastening-pivoted to the piece D and having a hook E, side lugs *e*, and handle H1, and the spring-catch I, all substantially as and for the purposes set forth.

4. In a horse-collar, the combination of the side pieces A, the hames, the cappiece, and the bolts hinged at their inner ends to the cap-piece, extended thence outward through the hames and secured, all substantially as and for the purposes set forth.

**449,573. HEEL-EXPANDER FOR HORSES.** GEORGE T. CHAPMAN, White Plains, assignor of one-half to William Harvey Merritt, New York, N. Y. Filed Aug. 8, 1889. Serial No. 320,170. (No model.)

*Claim.*—1. The graduated U-shaped spring-expander having the upright legs at the heel and laterally-projecting feet thereof, and being pointed at the extremities of the feet, substantially as described.

2. The combination, with a horseshoe, of a graduated U-spring hoof-expander interposed between the upper inner margin of the shoe and the roof to which the shoe is attached, and having the hooked-tongue expansion of the middle to engage the toe of the shoe, substantially as described.

3. The combination, with a horseshoe, of a graduated U-spring hoof-expander interposed between the upperi nner margin of the shoe and the hoof to which the shoe is attached, and having the notches of the heel extremities and the hooked tongue-extension of the middle, substantially as described.

4. The graduated U-spring hoof-expander having the tongue-entension of the middle and the perforations for nailing said spring to the hoof, substantially as described.

5. The graduated U-spring expander having the tongue-extension of the middle and the feet and legs at the heel and the perforations for nailing the spring to the hoof, substantially as described.

**449,622. WATERING-TROUGH FOR STOCK-CARS.** JOHN C. HICKS, Minneapolis, Minn., assignor, by mesne assignments, to the Hicks Stock Car Company, Chicago, Ill. Filed Mar. 18, 1890. Serial No. 344,294. (No model.)

*Claim.*—1. In a stock-car, watering-troughs located within the spaces between adjacent side posts or stanchions and supported removably therebetween, in combination with removable double-deck sections located when in position for use substantially in the same horizontal plane with said troughs, the outer longitudinal edges of said deck-sections being wholly inside said side posts, whereby spaces are left between said side posts which are not covered by said deck-sections, and covers or lids pivotally connected to the frame-work of the car, which swing over said troughs and form shields thereto, said covers or lids also bridging said spaces between the side posts along the longitudinal edges of said double-deck sections, substantially as set forth.

2. In a stock-car, watering troughs located within the spaces between adjacent side posts or stanchions and supported removably therebetween, in combination with covers or lids which swing over said troughs and which are pivotally connected to the frame-work of the car, substantially as set forth.

**449,228. ANIMAL-CHUTE.** LEWIS H. MYERS and SAMUEL H. ARMSTRONG, Canton, Ill. Filed Mar. 8, 1890. Serial No. 343,211. (No model.)

*Claim.*—1. The animal chute or gangway having its sides hinged to its floor, and means for locking the same in an upright position with relation to said floor, consisting of supporting-legs pivotally secured to the floor and a button on each of the legs adapted to engage a side, substantially as set forth.

2. The animal chute or gangway having its sides hinged to its floor, and means for locking the same in an upright position with relation to said floor, consisting of supporting-legs pivotally secured to the floor and a button on each of the legs adapted to engage a side, said side having uprights adapted to receive a leg between them, substantially as set forth.

3. The animal chute having inwardly-folding hinged sides and having legs provided with pivots which have their axes transverse to the side pieces of the floor and in the same plane therewith, substantially as set forth, whereby the sides can be folded down upon the floor and the legs placed parallel with the same.

**449,532. HORSE-PROTECTOR.** SAMUEL P. HODGEN, Pittsfield, Ill. Filed July 8, 1890. Serial No. 358,119. (No model.)

*Claim.*—1. In a protector of the class described, the combination, with a back-brace formed in sections, each of which is hollow, and one of said sections being adapted to slide within the other, of a series of pairs of clips secured to the upper sides of the brace and a series of arms of spring-wire bent to form loops and sprung between each pair of clips, substantially as specified.

2. In a protector of the class described, the back-band of a harness, a central brace provided with protector-supporting arms, a protecter mounted on the arms, and a clasp removably secured to the brace and to the said backband, substantially as specified.

3. In a protector of the class described, the combination, with a back-section and the shoulder-braces, each of said braces being formed of two sections telescopically connected, the front section of the shoulder-brace being provided with perforations and the rear section with a bolt for taking into anyone of said perforations, of a series of transverse arms, a protector or cover mounted on the arms, and a loose link connecting the ends of the braces, substantially as specified.

4. In a protector of the class described, the combination, with a pair of hame-sections having screw-eyes, of a central back-section adapted to be secured to the harness and provided with protector-supporting arms, a curved rectangular frame, the side bars of which are provided with offsets for engaging the eyes, and a cover supported by the arms and said rectangular frame, substantially as specified.

5. In a protector of the class described, the combination, with the main central brace, the secondary brace, the link connecting the two, and the arms projecting from said braces, of the crown-strap of the bridle, provided with opposite pairs of clips, a spring-wire frame mounted in the clips and having opposite upturned end portions forming a bonnet-frame, a bonnet mounted on said frame, and a cover or protector mounted on the arms, substantially as specified.

6. The protector-brace provided with lateral supporting-arms, the cover mounted on the same, and the herein-described fastening device, the same consisting of the base-plate provided with the depending T-lug for connecting with the back-strap, the upwardly-diverting leaves, one of which terminates in curved prongs, and a leaf hinged to the opposite prong and oppositely notched and adapted to be sprung over and between said prongs, substantially as specified.

7. The back-strap of a harness, provided near its front and rear ends with clasps 9, the herein-described protector-frame, the same comprising a central longitudinal main section and a series of laterally-disposed arms 6, and the protector mounted over the arms, substantially as specified.

**449,621. DOUBLE-DECK STOCK-CAR.** Bohn C. Hicks, Minneapolis, Minn., assignor, by mesne assignments, to the Hicks Stock Car Company, Chicago, Ill. Filed Feb. 17, 1890. Serial No. 340,786. (No model.)

*Claim.*—1. In a stock-car, the side posts thereof, watering troughs located between said side posts, and a removable upper deck located when in position for use in substantially the same horizontal plane as said troughs, in compination with brackets pivoted between each pair of side posts, the pivotal points of said brackets being located above said troughs and said brackets being so pivoted that their lower ends swing outwardly, each pair of brackets carrying on their lower end a plank or lid which when said brackets occupy their normal position serves both as a cover for the trough therebeneath and as an extension between the side posts of the upper deck, said brackets being capable of swinging outwardly far enough to uncover said troughs, substantially as set forth.

2. In a stock-car, the side posts thereof, watering-troughs located between said side posts, and a removable upper deck located when in position for use in substantially the same horizontal plane as said troughs, in combination with a pair of brackets pivoted between each pair of side posts, the pivotal points of said brackets being located above said troughs and said brackets being so pivoted that their lower ends swing outwardly, each pair of brackets carrying at their lower ends a plank or lid which when said brackets occupy their normal position serves both as a cover for the trough therebeneath and as an extension between the sideposts of the upper deck, said brackets being capable of swinging outwardly far enough to uncover said troughs, and said brackets carrying slats *i i*, which constitute when the brackets are in their normal position part of the exterior boarding of the car, substantially as set forth.

3. In a stock-car, the side posts thereof, watering-troughs located between said side posts, and a removable upper deck located when in position for use in substantially the same horizontal plane as the said watering-troughs, in combination with a pair of brackets pivoted between each pair of side posts, the pivotal points of said brackets being located above said troughs, and said brackets being so pivoted that their lower ends swing outwardly, each pair of brackets carrying at their lower ends a plank or lid which when said brackets occupy their normal position serves both as a cover for the trough therebeneath and as an extension between said side posts of the upper deck, said brackets being capable of swinging outwardly far enough to uncover said troughs, and longitudinal slats *i i*, extending along the sides of the car, said slats being secured to the outer edges of a plurality of said pairs of brackets, whereby said slats perform a threefold function, to wit: first, they constitute when the brackets are in their normal position a portion of the side walls of the car; second, they enable a plurality of the pairs of brackets to be operated simultaneously, and, third, they constitute stops to prevent the inward swinging of the brackets, substantially as set forth.

4. In a stock-car, the side posts thereof and watering-troughs located between said side posts, in combination with outwardly-swinging covers or lids, one for each of said troughs, each cover or lid being pivotally connected with and between two of the side pofts, a longitudinal shaft journaled along the side of the car and common to a plurality of said lids or covers, a plurality of crank-arms on said shaft, and links connecting said crank-arms and said lids or covers, substantially as set forth.

5. In a stock-car, the side posts on opposite sides thereof, watering-troughs located between said side posts on both sides of the car and a removable upper deck located when in position for use in substantially the same horizontal plane as said troughs, in combination with a pair of brackets pivoted between each pair of side posts, the pivotal points of said brackets located above said troughs and said brackets being so pivoted that their lower ends swing outwardly, each pair of brackets carrying at their lower ends a plank or lid which when said brackets occupy their normal position serves both as a cover for the trough therebeneath and as an extension between said side posts of the upper deck, said brackets being capable of swinging outwardly far enough to uncover said troughs, longitudinal slats *i i*, extending along each side of the car, said slats on each side of the car being secured to the outer edges of a plurality of said pairs of brackets, whereby said slats perform a threefold function, to wit: first they constitute when the brackets are in their normal position a portion of the side walls of the car; second, they enable a plurality of the pairs of brackets to be operated simultaneously, and, third, they constitute stops to prevent the inward swinging of the brackets, a longitudinal shaft G1, on each side of the car, a plurality of crank-arms *g*1 on each shaft, a plurality of links H, connecting said crank-arms and said brackets, an operating-wheel *f* on the end of the car, crank-arms *g* on the end of each shaft, and links G, connecting said crank-arms *g* with said wheel *f*, substantially as set forth.

**449,368.** HOOF-PAD. JOHN KRESS, New York, N. Y. Filed Jan. 7, 1891. Serial No. 376.961. (No model.)

*Claim.*—1. A hoof-pad for horses, composed of an elastic pad having transverse ribs at its under side, projecting lugs out of its front end and sides, a cavity at its middle portion, and a longitudinal pin secured to the pad and extended centrally through the cavity, substantially as set forth.

2. A hoof-pad for horses, consisting of a pad of elastic material having transverse ribs at its under side, an arc-shaped plate embedded into the front part of the pad and provided with projecting lugs at the front and sides, a cavity at the middle portion of the pad, and a longitudinal pin embedded in the pad and extending centrally through said cavity, substantially as set forth.

## Figures and Tables

**Figure f1:**
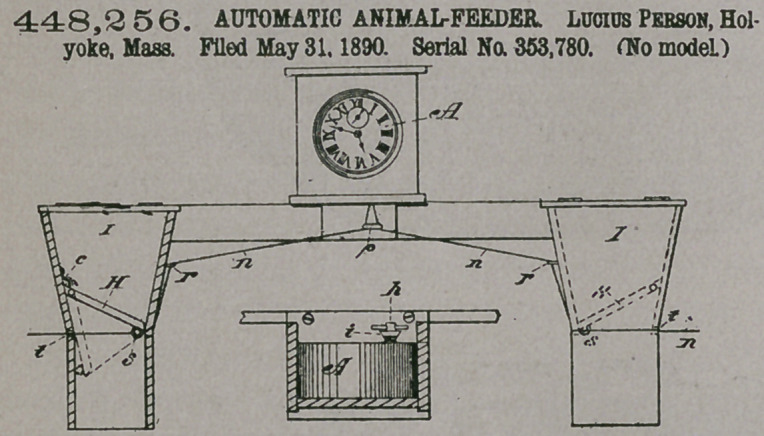


**Figure f2:**
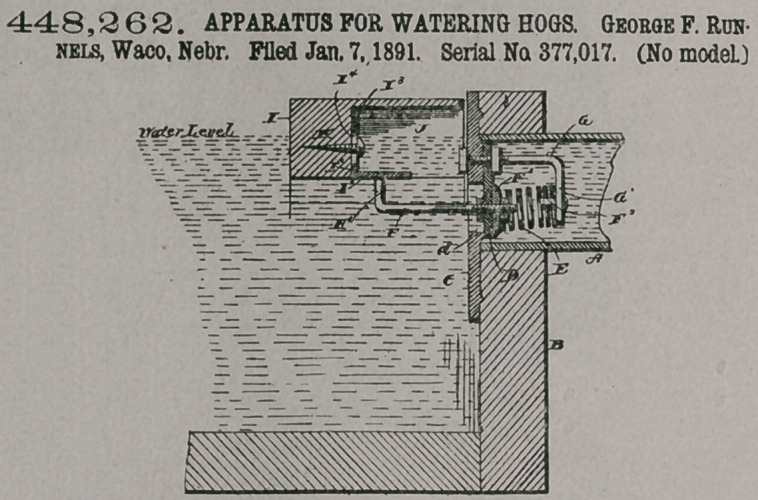


**Figure f3:**
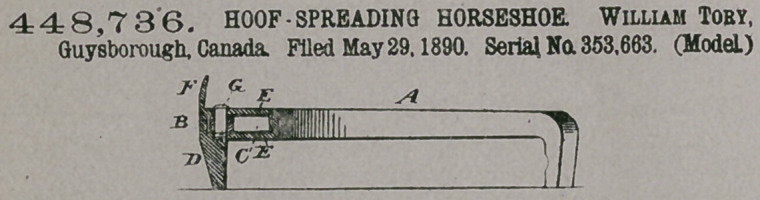


**Figure f4:**
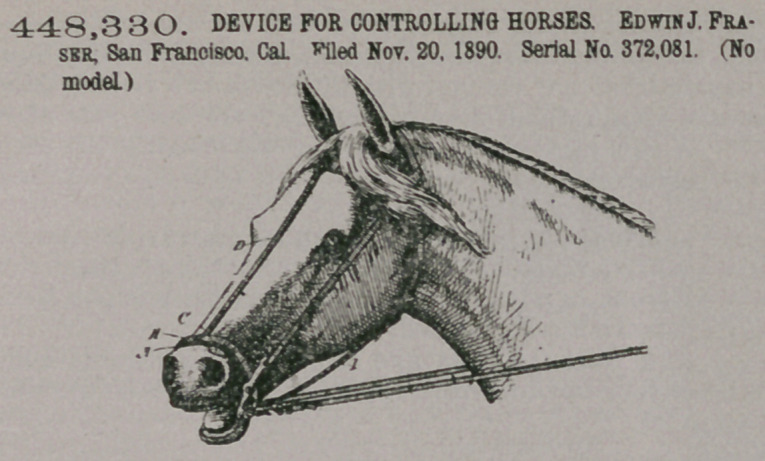


**Figure f5:**
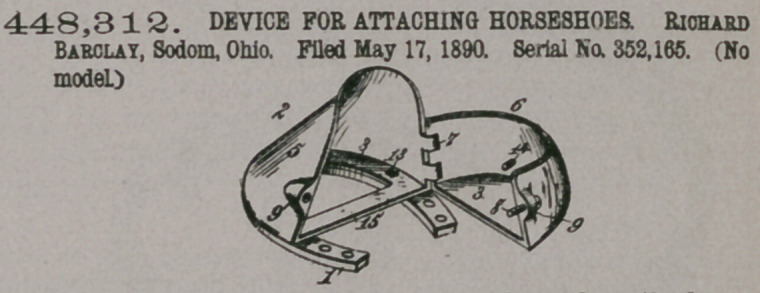


**Figure f6:**
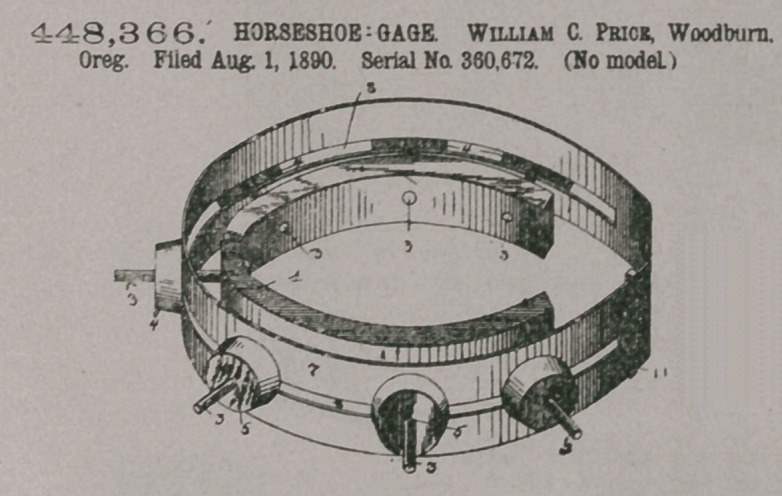


**Figure f7:**
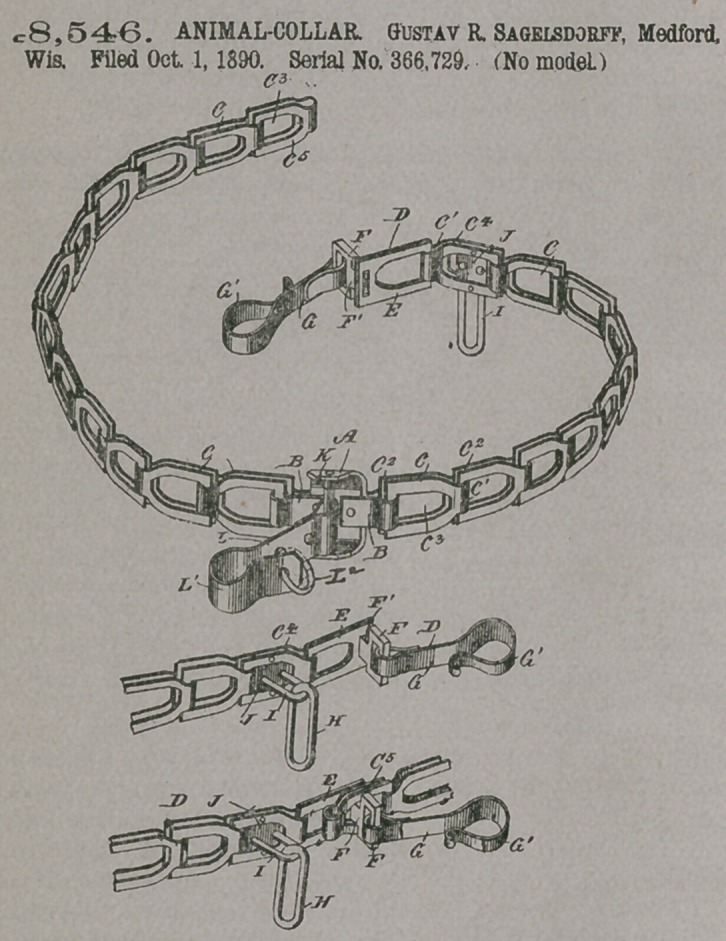


**Figure f8:**
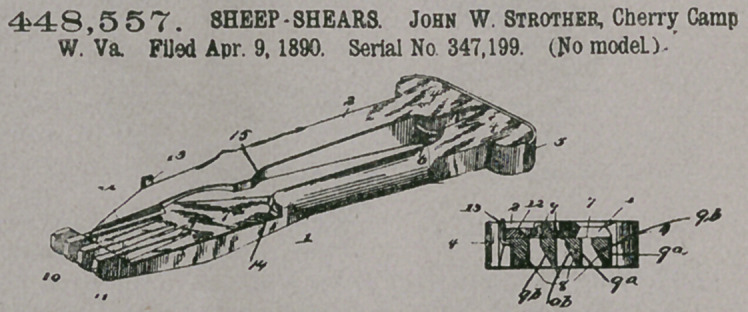


**Figure f9:**
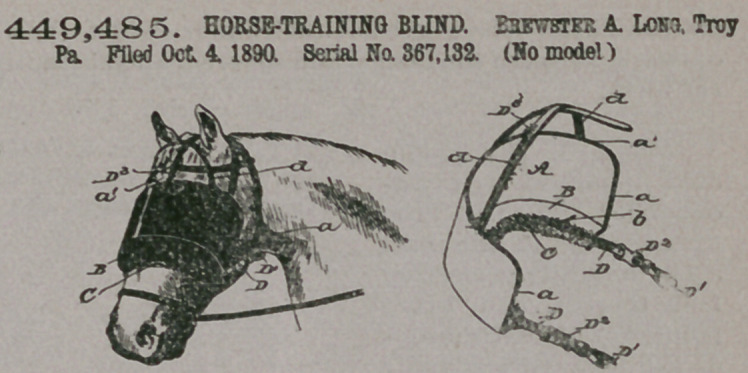


**Figure f10:**
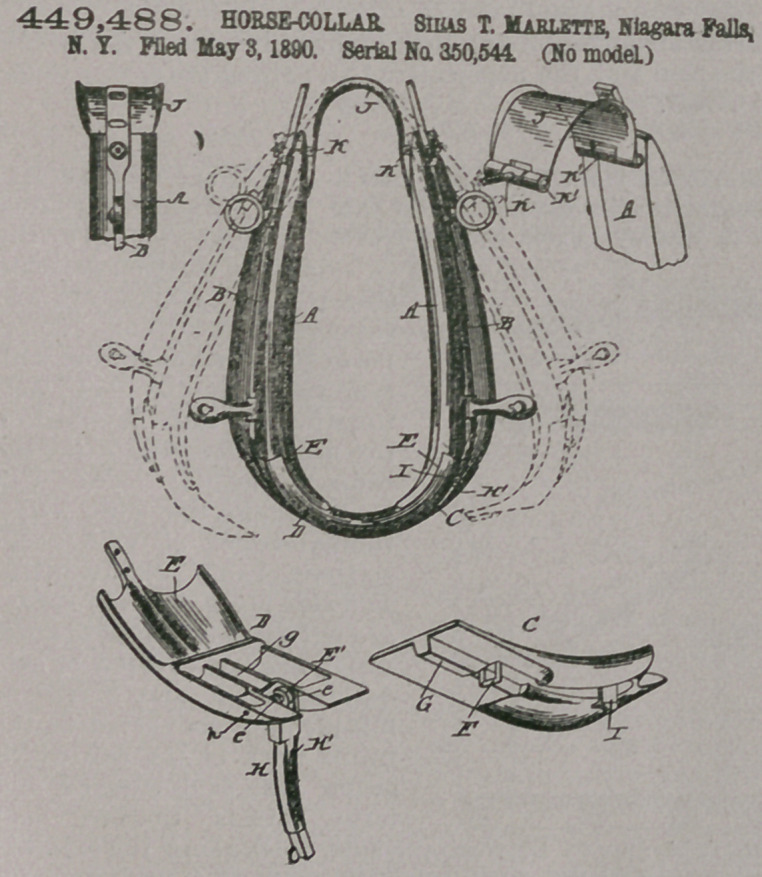


**Figure f11:**
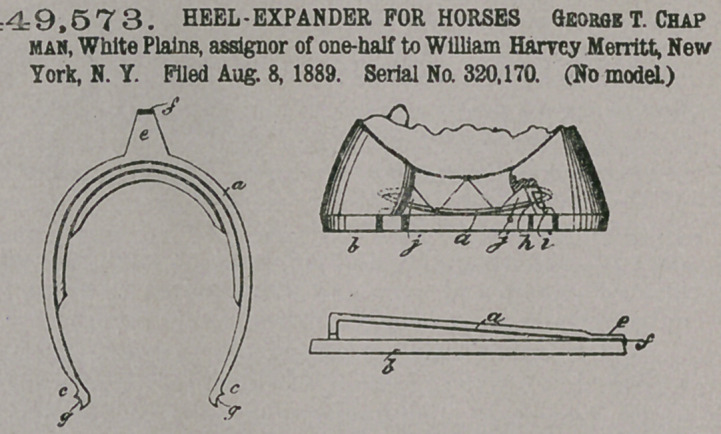


**Figure f12:**
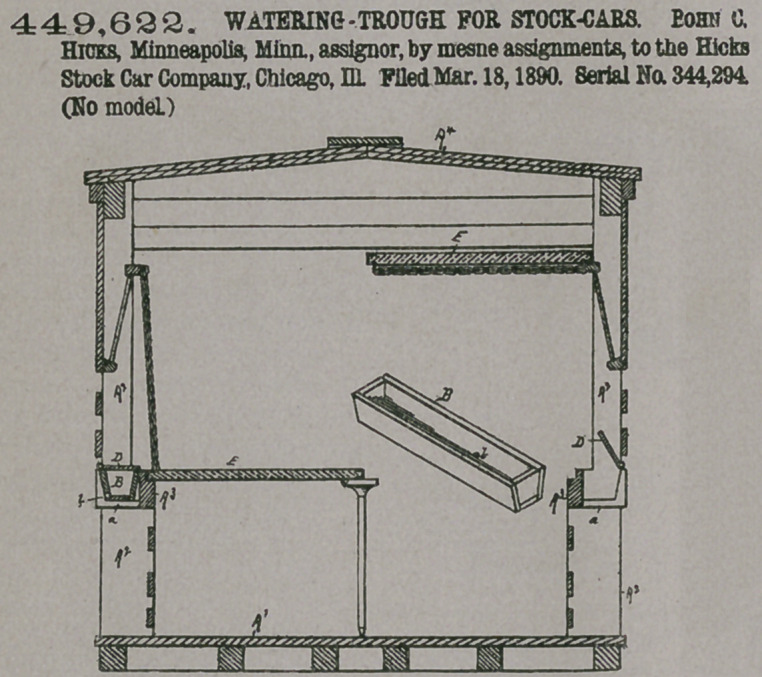


**Figure f13:**
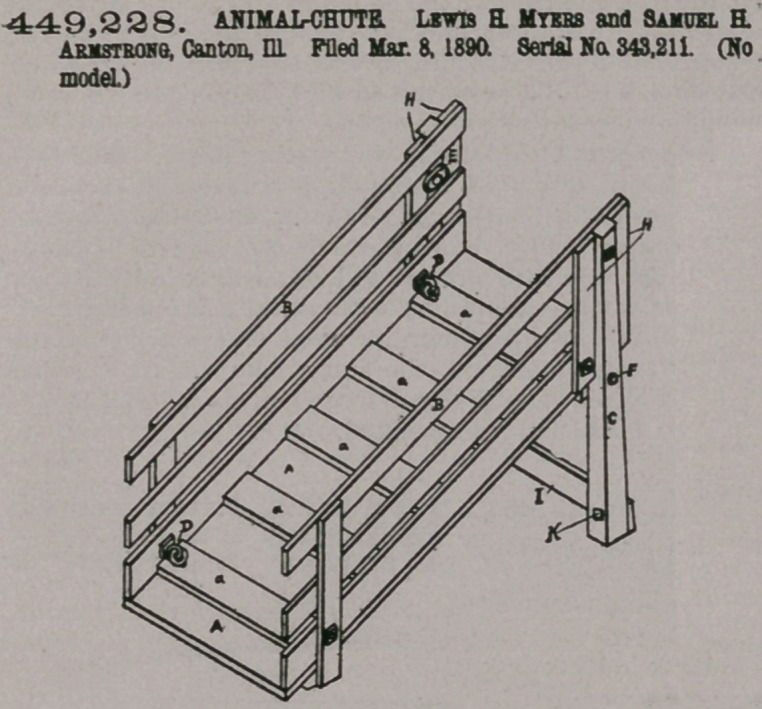


**Figure f14:**
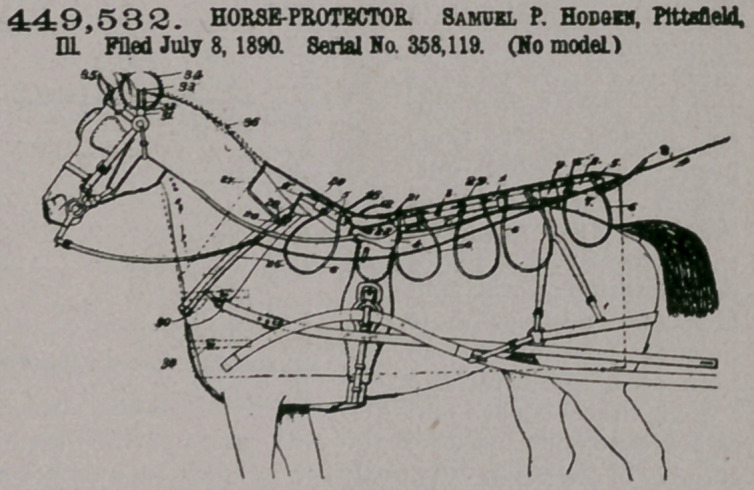


**Figure f15:**
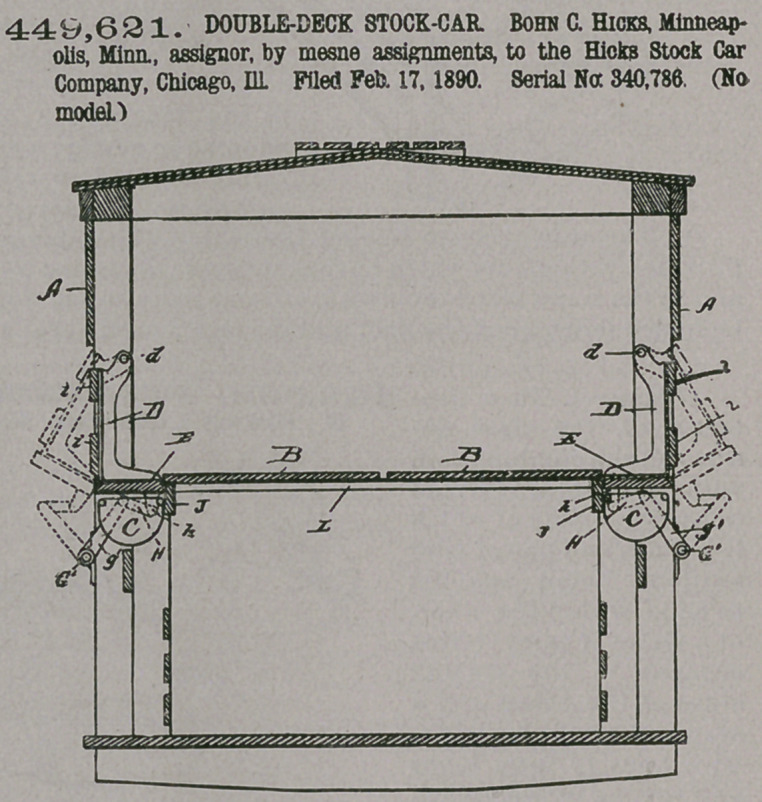


**Figure f16:**